# Mechanisms Underlying Sensory Nerve-Predominant Damage by Methylmercury in the Peripheral Nervous System

**DOI:** 10.3390/ijms252111672

**Published:** 2024-10-30

**Authors:** Tsuyoshi Nakano, Eiko Yoshida, Yu Sasaki, Shigekatsu Kazama, Fumika Katami, Kazuhiro Aoki, Tomoya Fujie, Ke Du, Takato Hara, Chika Yamamoto, Tsutomu Takahashi, Yasuyuki Fujiwara, Komyo Eto, Yoichiro Iwakura, Yo Shinoda, Toshiyuki Kaji

**Affiliations:** 1 Faculty of Pharmaceutical Sciences, Tokyo University of Science, 2641 Yamazaki, Noda 278-8510, Chiba, Japan; tsuyoshi.nakano@phar.toho-u.ac.jp (T.N.); yoshida3881@criepi.denken.or.jp (E.Y.); t-fujie@rs.tus.ac.jp (T.F.); 2 Faculty of Pharmaceutical Sciences, Toho University, 2-2-1 Miyama, Funabashi 274-8510, Chiba, Japan; takato.hara@phar.toho-u.ac.jp (T.H.); yamamoto@phar.toho-u.ac.jp (C.Y.); 3 Sustainable System Research Laboratory, Central Research Institute of Electric Power Industry, 1646 Abiko, Abiko 270-1194, Chiba, Japan; 4 School of Pharmacy, China Medical University, No.77 Puhe Road, Shenyang North New Area, Shenyang 110122, China; kdu@cmu.edu.cn; 5 School of Pharmacy, Tokyo University of Pharmacy and Life Sciences, 1432-1 Horinouchi, Hachioji 192-0392, Tokyo, Japan; tsutomu@toyaku.ac.jp (T.T.); yasuyuki@toyaku.ac.jp (Y.F.); 6 Health and Nursing Facilities for the Aged, Jushindai, Shinwakai, 272 Ikurakitakata, Tamana 865-0041, Kumamoto, Japan; shinwa@drive.ocn.ne.jp; 7 Research Institute for Biomedical Sciences, Tokyo University of Science, 2641 Yamazaki, Noda 278-8510, Chiba, Japan; iwakura@rs.tus.ac.jp

**Keywords:** methylmercury, Minamata disease, sensory nerve, peripheral nerve

## Abstract

Sensory disturbances and central nervous system symptoms are important in patients with Minamata disease. In the peripheral nervous system of these patients, motor nerves are not strongly injured, whereas sensory nerves are predominantly affected. In this study, we investigated the mechanisms underlying the sensory-predominant impairment of the peripheral nervous system caused by methylmercury. We found that the types of cell death in rat dorsal root ganglion (DRG) neurons caused by methylmercury included apoptosis, necrosis, and necroptosis. Methylmercury induced apoptosis in cultured rat DRG neurons but not in anterior horn neurons or Schwann cells. Additionally, methylmercury activated both caspase 8 and caspase 3 in DRG neurons. It increased the expression of tumor necrosis factor (TNF) receptor-1 and the phosphorylation of receptor-interacting protein kinase 3 (RIP3) and mixed-lineage kinase domain-like pseudokinase (MLKL). The expression of TNF-α was increased in macrophage-like RAW264.7 cells by methylmercury. The increase was suggested to be mediated by the NF-κB pathway. Moreover, methylmercury induced neurological symptoms, evaluated by a hindlimb extension response, were significantly less severe in TNF-α knockout mice. Based on these results and our previous studies, we propose the following hypothesis regarding the pathogenesis of sensory nerve-predominant damage by methylmercury: First, methylmercury accumulates within sensory nerve neurons and initiates cell death mechanisms, such as apoptosis, on a small scale. Second, cell death triggers the infiltration of macrophages into the sensory fibers. Third, the macrophages are stimulated by methylmercury and secrete TNF-α through the NF-κB pathway. Fourth, TNF-α induces cell death mechanisms, including necrosis, apoptosis through the caspase 8/3 pathway, and necroptosis through the TNFR1-RIP1-RIP3-MLKL pathway, activated by methylmercury in sensory neurons. Consequently, methylmercury exhibits potent cytotoxicity specific to the DRG/sensory nerve cells in the peripheral nervous system. This chain of events caused by methylmercury may contribute to sensory disturbances in patients with Minamata disease.

## 1. Introduction

Minamata disease (MD) is a neurological disorder caused by methylmercury poisoning. Methylmercury-induced damage extends to the cerebrum, cerebellum, and peripheral nerves. Lesions in the cerebrum are localized along the deep sulci and fissures in the cerebral cortex, such as the calcian fissures, in patients with MD [1]. Methylmercury-induced edematous changes were observed in the white cortex of the cerebrum, suggesting that damage in neuronal cells along deep sulci was due to cortex swelling-induced ischemia in the narrow space in the cerebrum (“Edema hypothesis”). We revealed that the mechanisms underlying edematous changes include the activation of the vascular endothelial growth factor system, which increases vascular permeability in a paracrine manner between brain endothelial cells and pericytes [2]. Additionally, methylmercury promotes hyaluronan secretion from vascular endothelial cells and pericytes, contributing to the progression of edematous change [3].

Specific injury to the granule cell layer occurs in the cerebellum, which causes cerebellar ataxia. In the mild to moderate stages, Purkinje cells are relatively preserved, whereas granule cells just under the Purkinje cell layer are selectively injured, resulting in central cerebellar atrophy. A histological observation of the rat cerebellum suggests that a small degree of cell death in granular cells triggers cytotoxic T lymphocytes and macrophages to invade the granule cell layers and cause damage there [4]. We postulate that cytotoxic T lymphocytes and macrophages are involved in the death of granule cells through TNF-α and perforin/granzyme B secretions, respectively. We propose an “inflammatory hypothesis” regarding the pathogenesis of granule cell-specific damage in the cerebellum of patients with MD.

Sensory disturbances are characteristic of MD. Pathologically, this sensory disturbance is postulated to be central, based on damage to the postcentral gyrus and other parts of the cerebrum, or peripheral, based on peripheral sensory nerve damage [5]. Importantly, marked axonal degeneration of the sensory nerves was observed in the peripheral nerves of patients with MD and experimental animals exposed to methylmercury, whereas such degeneration was not observed in the motor nerves [6,7,8]. However, the molecular and cellular bases of the peripheral sensory nerve lesions observed in patients with MD have not been fully elucidated, and there remain many unknown aspects regarding the sensory disturbances caused by peripheral sensory nerve-predominant damage.

Recently, we reported that cultured rat dorsal root ganglion (DRG) cells were highly susceptible to methylmercury compared to anterior horn cells (AHCs) and Schwann cells. This was due to a higher accumulation of intracellular methylmercury based on the higher expression of L-type amino acid transporter 1 and the lower expression of multidrug resistance-associated protein 2 [9]. This may be a part of the mechanisms underlying the predominant damage caused by methylmercury in sensory nerve cells in the peripheral nervous system. Additionally, our previous studies revealed that macrophages infiltrated the sensory nerve fibers with degeneration of sensory neurons and sensory impairment, and there was no noticeable change in the motor fibers in rats [10]. Moreover, gene expression profiles in the DRG neurons of rats suggested caspase 8 and TNF-α as possible signaling molecules that can mediate cell death in sensory nerve cells [11]. In the present study, we characterized the types of cell death in cultured rat DRG neurons and the signaling pathways involved in cell death upon methylmercury exposure. Furthermore, the involvement of TNF-α was also investigated.

## 2. Results

### 2.1. Types of DRG Cell Death Caused by Methylmercury Include Apoptosis, Necrosis, and Necroptosis

**Figure 1:**
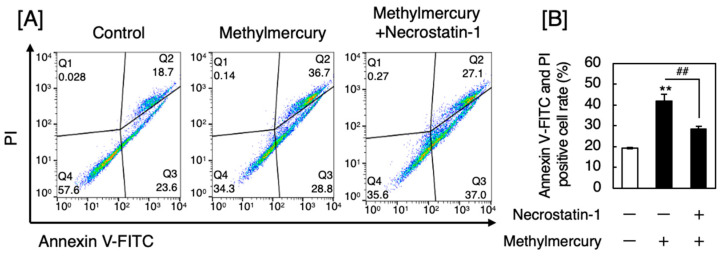
Annexin V-fluorescein isothiocyanate (FITC)/PI flow cytometry analysis of DRG neurons after treatment with methylmercury for 24 h. (**A**) Fluorescence-activated cell sorting (FACS) using flow cytometry of cultured rat DRG neurons pretreated with or without necrostatin-1 (1 µM) for 1 h and then treated with methylmercury (0.5 µM) for 24 h. (**B**) Quantitative analysis of the flow cytometry data. Values are means ± standard error (S.E.) of three technical replicates. ** Significantly different from the control, *p* < 0.01; ## significantly different from the treatment with methylmercury alone, *p* < 0.01. The data in A are representative of several independent experiments.

First, we investigated the type of death in sensory nerve cells exposed to methylmercury. The sacral vertebrae were resected from rats, and DRG neurons, which are relay points for sensory information, were separated, cultured, and treated with methylmercury. Cell death was analyzed using fluorescence-activated cell sorting (FACS). Here, apoptotic cells were positive for annexin V, while necrotic or necroptotic cells were positive for both annexin V and propidium iodate (PI). Methylmercury caused both apoptosis and necrosis/necroptosis in DRG neurons (Figure 1A). Necrostatin-1, which suppresses necroptosis by inhibiting receptor-interacting protein kinase 1 (RIP1), significantly and partly reduced the necrotic/necroptotic DRG neurons (Figure 1B), suggesting that methylmercury causes necrosis, necroptosis, and apoptosis.

### 2.2. Intracellular Signal Pathways Mediating Apoptosis and Necroptosis in DRG Neurons

**Figure 2:**
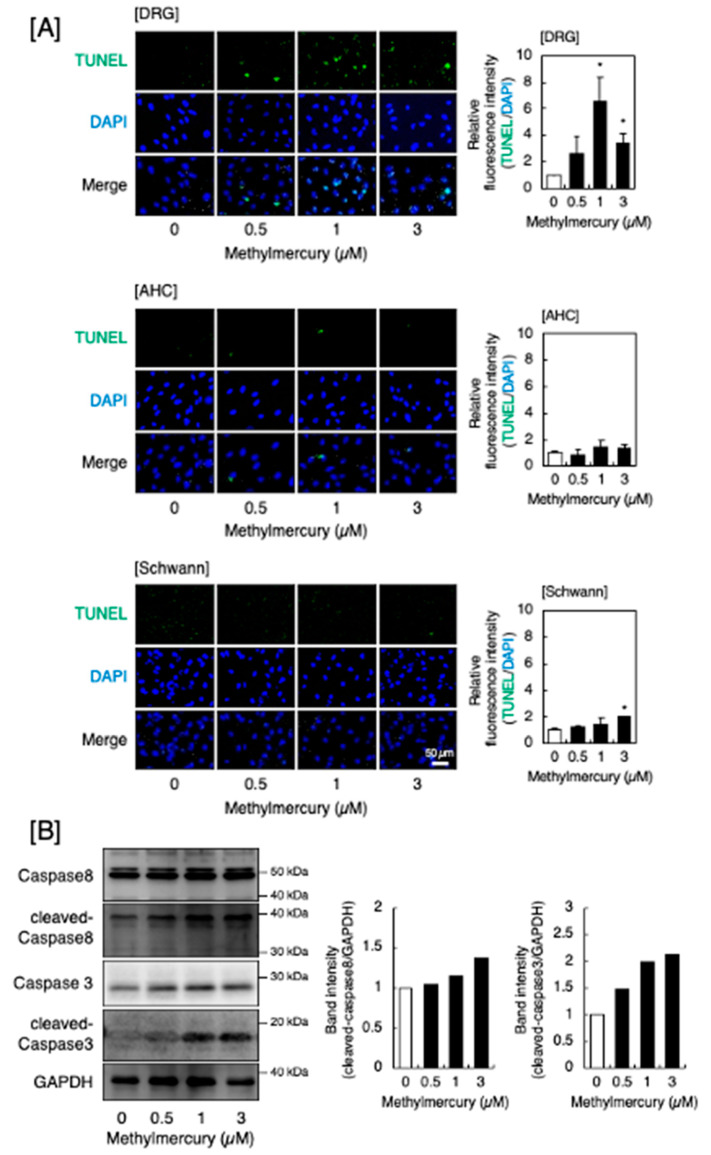
Apoptosis induced by methylmercury. (**A**) Representative images of the TUNEL assay (**left** panels, 100× magnification) and the quantitative analysis (**right** panels) of cultured DRG neurons (**upper** panels), AHCs (**middle** panels), and Schwann cells (**lower** panels) after treatment with methylmercury (0.5, 1, and 3 µM) for 24 h. Values are means ± S.E. of three technical replicates. Significantly different from the corresponding control, * *p* < 0.05. (**B**) Activation of caspase 8 and caspase 3 by methylmercury in DRG neurons. The cells were treated with methylmercury (0.5, 1, and 3 µM) for 24 h, and the expression of caspase 8, cleaved caspase 8, caspase 3, and cleaved caspase 3 was determined by Western blot analysis. Glyceraldehyde-3-phosphate dehydrogenase (GAPDH) was used as an internal control. The representative Western blot of at least two experiments is shown.

As necrosis occurs without the involvement of intracellular signaling pathways, we examined the signaling pathways that induce apoptosis and necroptosis. Figure 2A shows the occurrence of methylmercury-induced apoptosis in DRG neurons, AHCs, and Schwann cells. Apoptotic cells were detected by terminal deoxynucleotidyl transferase dUTP nick end labeling (TUNEL) staining, and methylmercury significantly increased the number of apoptotic cells. Apoptosis was also observed in AHCs and Schwann cells, but to a much lesser extent than in DRG neurons. Methylmercury increased the cleaved caspase 8 and cleaved caspase 3 levels in DRG neurons (Figure 2B), indicating that methylmercury-induced apoptosis in DRG neurons was mediated by the caspase 8–caspase 3 pathway. Full-length caspases 8 and 3 were not decreased despite the increase in cleaved caspase 8 and 3. We performed an additional experiment to check the expression of *caspase 3* and *caspase 8* mRNAs and found that the expression was significantly increased by methylmercury. Methylmercury may increase the expression of full-length caspases 8 and 3.

**Figure 3:**
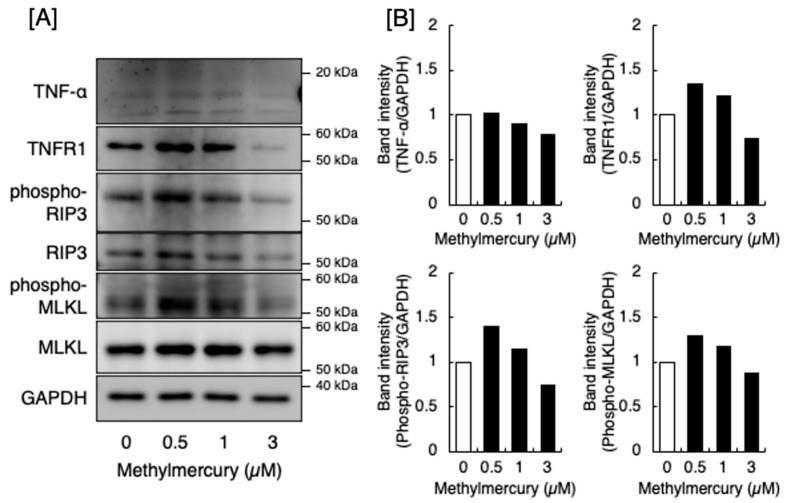
Expression of signaling molecules in the downstream pathway of TNF-α-activated TNFR1 involved in the necroptosis of DRG neurons. (**A**) Expression of TNF-α, TNFR1, phosphorylated RIP3, RIP3, phosphorylated MLKL, and MLKL. The molecules were detected by Western blot analysis. GAPDH was used as an internal control. The data shown are representative of at least two experiments. (**B**) Quantitative analysis of the Western blot data.

In contrast, TNF-α can induce necroptosis through RIP3 and RIP1 and the consequent phosphorylation of mixed-lineage kinase domain-like pseudokinase (MLKL) by RIP3, which is a downstream pathway of TNF-α-activated TNF receptor-1 (TNFR1) [12]. As shown in Figure 3, methylmercury increased the expression of p-RIP3 and p-MLKL in DRG neurons. Methylmercury at 3 µM decreased the expression of p-RIP3 and p-MLKL. This may be due to nonspecific cell damage caused by methylmercury. In other words, the cell death of DRG neurons caused by methylmercury at 3 µM may be necrosis and apoptosis rather than necroptosis. TNFR1 expression also increased following methylmercury treatment. However, the expression of TNF-α was unchanged by methylmercury, suggesting that methylmercury induces necroptosis in DRG neurons by activating the TNFR1-RIP1-RIP3-MLKL pathway. However, TNF-α, which activates this pathway, was assumed to be derived from other cell type(s).

### 2.3. Methylmercury Induced the Synthesis of TNF-α in Macrophage-like RAW264.7 Cells

**Figure 4:**
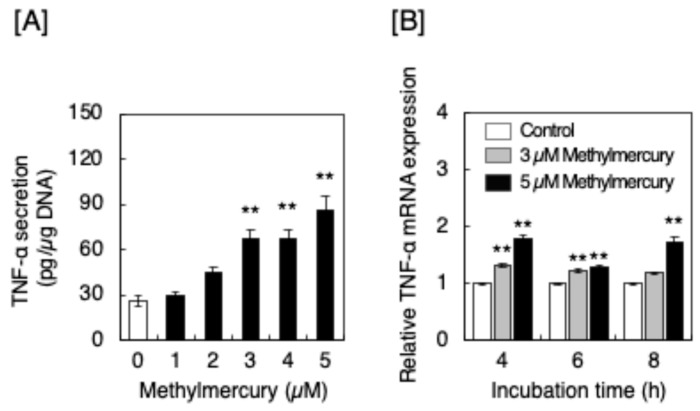
Secretion and expression of TNF-α in macrophage-like RAW264.7 cells after treatment with methylmercury. (**A**) Secretion of TNF-α from RAW264.7 cells treated with methylmercury (1, 2, 3, 4, and 5 µM) for 12 h. Values are means ± S.E. of three technical replicates. ** Significantly different from the control, *p* < 0.01. (**B**) Expression of *TNF-α* mRNA in RAW264.7 cells treated with methylmercury (3 and 5 µM) for 4, 6, and 8 h. Values are means ± S.E. of three technical replicates. ** Significantly different from the corresponding control, *p* < 0.01.

As macrophages accumulated in the sensory nerve fibers of rats after exposure to methylmercury [10], we postulated that the TNF-α was derived from macrophages. Figure 4 shows the effect of methylmercury on the expression of TNF-α in cultured macrophage-like RAW264.7 cells. We found that methylmercury significantly increased the secretion of TNF-α from these cells and significantly elevated the expression of *TNF-α* mRNA, indicating that methylmercury promoted the synthesis of TNF-α in the macrophage-like cells.

**Figure 5:**
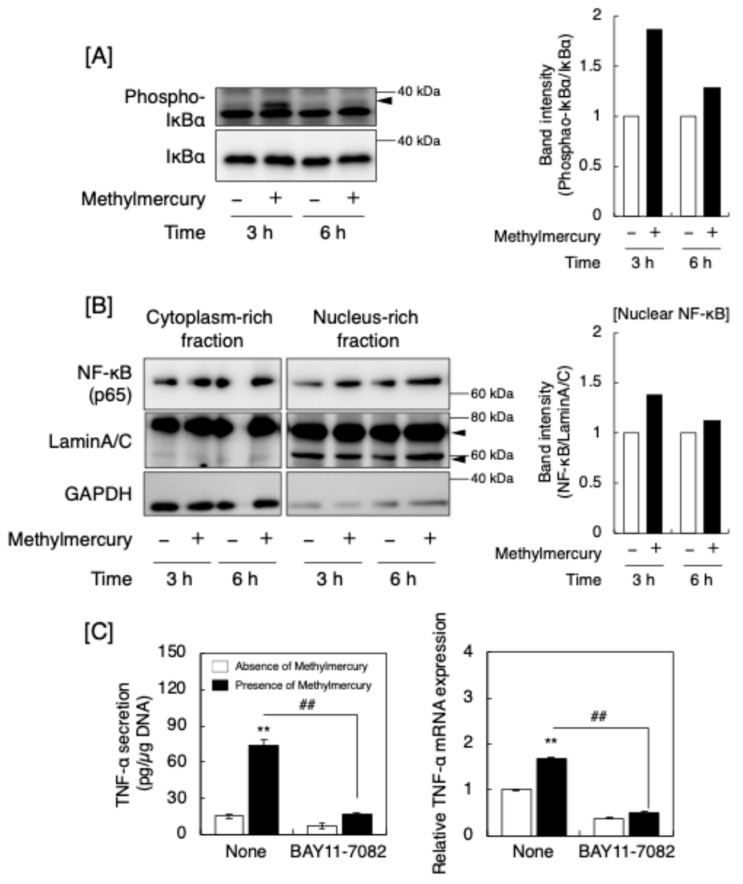
Involvement of NF-κB signaling in the induction of TNF-α by methylmercury in macrophage-like RAW264.7 cells. (**A**) Western blot images depicting the phosphorylation of IκBα. The arrowhead indicates the position of IκBα. (**B**) Nuclear translocation of NF-κB in the cells. Lamin A/C is an internal control for the nuclear fraction. Arrowheads indicate the position of Lamin A/C. (**C**) TNF-α secretion and mRNA levels upon treatment with an NF-κB inhibitor, BAY11-7082, and methylmercury. Values are means ± S.E. of three technical replicates. ** Significantly different from the corresponding control, *p* < 0.01; ## significantly different from the treatment with methylmercury alone, *p* < 0.01.

As the expression of macrophage TNF-α can be upregulated by mitogen-activated protein kinases (MAPKs) [13] and methylmercury can activate MAPKs such as p38 MAPK [14], we examined the phosphorylation of MAPKs, extracellular signal regulated kinase (ERK1/2), p38 MAPK, and c-jun N-terminal kinase (JNK) in RAW264.7 cells (Figure S1). Methylmercury increased MAPK phosphorylation (Figure S1A); however, a MAPK/ERK kinase inhibitor PD98059, a p38 MAPK inhibitor SB203580, and a JNK inhibitor SP600125 failed to inhibit the increase in TNF-α secretion and *TNF-α* mRNA levels in the cells (Figure S1B), suggesting that the MAPK pathway was activated but did not affect TNF-α synthesis in RAW264.7 cells exposed to methylmercury. Notably, nuclear factor-κB (NF-κB) can also mediate the upregulation of TNF-α synthesis in macrophages [15,16]. NF-κB consists of p65 and p50, which are bound to the inhibitor of κBα (IκBα). The phosphorylation of IκBα results in its proteasomal degradation, the nuclear translocation of the heterodimerized complex of p65 with p50, and the recruitment of the complex to κB binding sites in the promoter regions of target genes [17]. Methylmercury increased the phosphorylation of IκBα and the nuclear translocation of NF-κB (Figure 5A). Additionally, an NF-κB inhibitor, BAY11-7082, significantly reduced the induction of TNF-α at the protein and mRNA levels in RAW264.7 cells (Figure 5B). Furthermore, the siRNA-mediated knockdown of p65 also significantly reduced the methylmercury-induced increase in the secretion of TNF-α from these cells (Figure S2), suggesting that methylmercury induces TNF-α synthesis via the activation of NF-κB.

### 2.4. A Neurological Symptom Caused by Methylmercury Is Alleviated in TNF-α Knockout Mice

**Figure 6:**
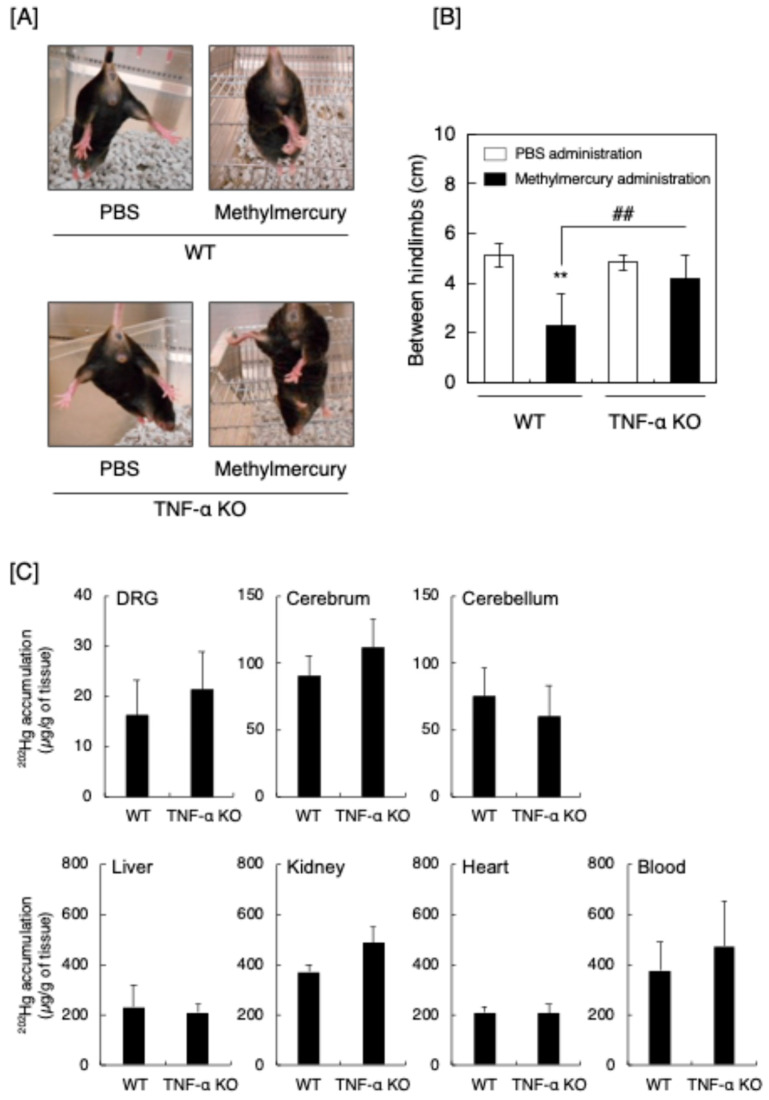
Hindlimb extension test of male wild-type (WT) and TNF-α KO mice. The mice were orally administered phosphate-buffered saline (PBS) or methylmercury (15 mg/kg/day) for 5 days, followed by no administration for 2 days, and then administration for 1 day. On day 9, which is one day after the last administration, the hindlimb extension test was performed. Separately, the accumulation of total mercury in the cerebrum, cerebellum, DRG, liver, kidney, heart, and blood was measured by inductively coupled plasma mass spectrometry (ICP-MS). (**A**) Representative features of the hindlimb extension test in mice. (**B**) Quantitative analysis of the hindlimb extension by measuring the distance between the hindlimbs. ** Significantly different from the corresponding control, *p* < 0.01; ## significantly different from the “methylmercury administration”, *p* < 0.01. (**C**) Accumulation of total mercury. Values are means ± S.E. of four mice. There was no significant difference between WT and TNF-α KO mice.

The above in vitro experiments indicated that the induction of TNF-α is involved in the predominant damage to sensory nerves and the consequent occurrence of sensory disturbance. Based on these results, neurological symptoms caused by methylmercury in TNF-α knockout (KO) mice were evaluated by the hindlimb extension test [18,19] (Figure 6). Methylmercury induced the hindlimb extension in wild-type (WT) mice; however, the extension was reduced in the TNF-α KO mice (Figure 6A). The degree of the extension was quantitatively analyzed by the distance between the hindlimbs, and the neurological symptoms caused by methylmercury were significantly reduced in the TNF-α KO mice (Figure 6B). However, the accumulation of total mercury was unchanged by TNF-α KO in the DRG, as well as in the cerebrum, cerebellum, liver, kidney, heart, and blood (Figure 6C).

## 3. Discussion

A significant part of the research on MD has focused on the toxicity of methylmercury to the brain [20,21] because patients with MD exhibit central nervous system symptoms, including ataxia, dysarthria, visual impairment, acoustic disturbance, and hypesthesia, with damage to the brain [22]. However, extensive DRG neuronal degeneration and damage to the sensory nerves caused by methylmercury have been observed in experimental animals [8,23,24]. Severe axonal degeneration, myelin degeneration, and the disappearance of DRG neurons have been observed histologically [5]. Previously, we found that methylmercury predominantly damages sensory nerves in the peripheral nervous system of rats, with the accumulation of macrophages within the sensory nerve fibers [10]. Recently, we showed that DRG cells are much more susceptible to methylmercury than AHCs and Schwann cells because of the higher expression of L-type amino acid transporter 1 and lower expression of multidrug resistance protein 2 [9]. Based on these and the results of the present study, we propose the following hypothesis regarding the pathogenesis of sensory nerve-predominant damage by methylmercury: First, methylmercury accumulates highly within sensory nerve cells and causes cell death on a small scale. Second, the cell death triggers the infiltration of macrophages into the sensory fibers. Third, the macrophages are stimulated by methylmercury and largely secrete TNF-α. Fourth, TNF-α induces cell death in sensory neurons. These sequential events occur selectively in sensory fibers because AHCs and Schwann cells are more resistant to methylmercury than DRG/sensory neurons. Consequently, methylmercury exhibits strong cytotoxicity specific to the DRG/sensory nerve cells in the peripheral nervous system.

Understanding the molecular basis of methylmercury toxicity in sensory neurons is important for understanding the mechanisms underlying sensory nerve-predominant damage caused by methylmercury. Methylmercury can induce necrosis [25] and apoptosis [25,26] in nerve cells, including DRG neurons [27]. Methylmercury causes necrosis or apoptosis in various cell types other than neurons, including peritoneal neutrophils [28], leukocytes [29], pancreatic β-cells [30], and myogenic cells [31]. However, little is known about the type of methylmercury-induced cell death in sensory neurons. The present study revealed that the types of cell death caused by methylmercury in DRG neurons include necrosis, apoptosis, and necroptosis. Although necrosis occurs without the activation of any intracellular signaling pathway, there are signaling pathways that mediate apoptosis and necroptosis. These pathways were investigated based on the results of our previous study [11]. Regarding the apoptosis of DRG neurons, the caspase 8/3 pathway was activated by methylmercury and appeared to be involved in apoptosis. Signaling pathways that mediate apoptosis by methylmercury in nerve cells depend on the cell type [32]; however, the caspase pathway appears to be one of the leading pathways [33,34,35,36]. In contrast, regarding necroptosis, the RIP1-RIP3-MLKL pathway, one of the downstream signaling cascades of TNFR1, activated by TNF-α, is the most understood signaling pathway [37]. Our previous study indicated that TNF-α can be a signal molecule that mediates cell death in sensory nerve cells [11]. In the present study, it was suggested that methylmercury activates the TNFR1-RIP1-RIP3-MLKL pathway and then causes necroptosis in DRG neurons, supporting the hypothesis that sensory nerve cell death caused by methylmercury is partly mediated by TNF-α.

The present data suggested that the source of TNF-α that activates the TNFR1-RIP1-RIP3-MLKL pathway to induce necroptosis in sensory nerve/DRG neurons is macrophages that infiltrate the sensory nerve fibers [10]. Previous studies by other researchers have shown a possible involvement of TNF-α in the neurotoxicity of methylmercury. For example, methylmercury induces TNF-α expression selectively in the brain of mice [38], although the mechanisms underlying the expression remain unclear. Importantly, TNF-α can be involved in cell death in the neural tissues after exposure to methylmercury. Methylmercury causes neuronal cell death by p38 MAPK phosphorylation-mediated TNF-α induction in microglia [39]. We also proposed a hypothesis that macrophages infiltrate the granule layer of the cerebellum after exposure to methylmercury and cause granule cell death, presumably by secreting TNF-α [4]. The induction of TNF-α expression is mediated by the NF-κB pathway [40]. In the present study, we demonstrated that methylmercury induces the expression of TNF-α by activating the NF-κB pathway in macrophage-like cells. As TNF-α activates the NF-κB pathway [41], methylmercury-induced TNF-α expression in macrophages may be amplified through the interaction between the action of TNF-α on the NF-κB pathway and the NF-κB signaling pathway that mediates the TNF-α expression.

**Figure 7:**
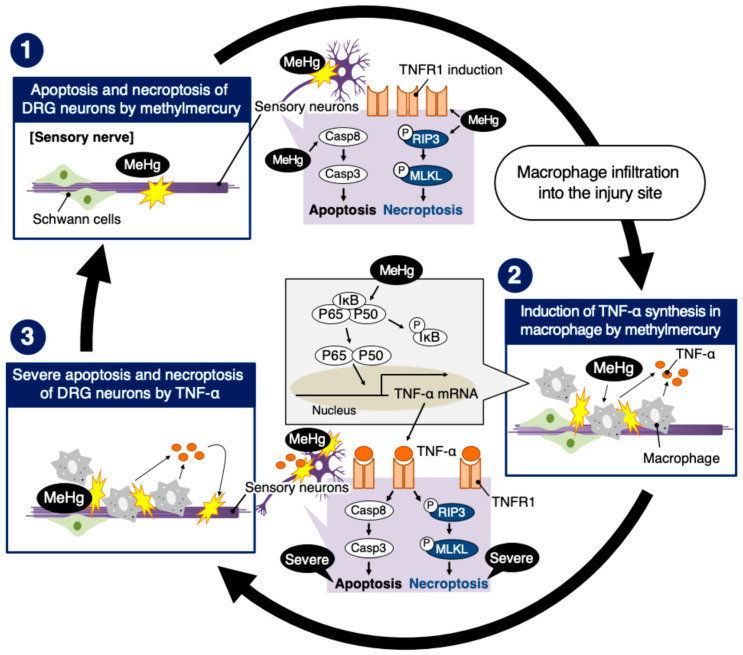
A hypothesis regarding the pathogenesis of sensory nerve-predominant damage in the peripheral nervous system after exposure to methylmercury. (1) As DRG neurons are susceptible to methylmercury due to the higher accumulation of methylmercury in the peripheral nerve system [11], early exposure to methylmercury causes sensory nerve cell death in the sensory fibers. (2) The cell death triggers the infiltration of macrophages into the sensory fibers [13]. The macrophages are stimulated by methylmercury and secrete TNF-α through the activation of the NF-κB pathway. (3) TNF-α induces apoptosis and necroptosis in sensory neurons by activating the caspase 8–caspase 3 pathway and the TNFR1-RIP1-RIP3-MLKL pathway, respectively. Although the possibility that that activation of the TNFR1-RIP1-RIP3-MLKL pathway was mediated by factors other than TNF-α such as TLR4 and Fas cannot be excluded, our data suggested that this pathway could be activated by TNF-α from macrophages infiltrating sensory fibers. Additionally, methylmercury causes necrosis in sensory neurons. The cell death of sensory neurons triggers macrophage infiltration in sensory fibers. These processes are cascaded and amplified, and methylmercury predominantly damages the sensory neurons.

We showed that the hindlimb extension by methylmercury was significantly reduced in TNF-α KO mice, suggesting that this neurological symptom is partly mediated by TNF-α. The fact that symptoms were independent of methylmercury accumulation partly supports this hypothesis. In other words, methylmercury neurotoxicity partly depends on the action of TNF-α in neural cells. It is postulated that the neurological symptoms in the hindlimbs of rodents are mainly due to damage in the cerebellum and/or sensory nerves. Our previous study [4] and present data support the hypothesis that methylmercury neurotoxicity toward the cerebellum and sensory nerves is mediated by TNF-α. We postulate that intracellular signaling pathways activated by methylmercury, which can be involved in the cell death of DRG neurons and TNF-α expression in macrophages, contribute to the neurological symptoms shown by hindlimb extension. It is possible that hindlimb extension was caused by damage to the cerebrum, cerebellum, and sensory nerve cells following exposure to methylmercury. However, we observed no change in the cerebrum or granule cell-specific damage with the infiltration of macrophages into the granule cell layers in the cerebellum of methylmercury-exposed rats [4]. Therefore, an assumption can be made that TNF-α-mediated neurotoxicity induced by methylmercury shares a similar pathogenesis in the cerebellum in the central nervous system and the sensory neurons in the peripheral nervous system. In summary, this study elucidates the pathogenesis of damage to sensory neurons in the peripheral nervous system after exposure to methylmercury. As shown in Figure 7, the pathogenesis involves three events: (1) cell death at a small scale in sensory neurons in the peripheral nervous system, (2) the infiltration of macrophages into the sensory nerve fibers, (3) the induction and secretion of TNF-α by the macrophages, and (4) cell death at a larger scale in sensory neurons caused by the TNF-α. Interestingly, methylmercury was involved in every event. The present data clearly indicate that there is a specific molecular basis to the death of DRG neurons/sensory neurons and the induction of TNF-α in macrophages after exposure to methylmercury. Therefore, TNF-α may be a therapeutic target molecule in certain stages of methylmercury poisoning.

## 4. Materials and Methods

### 4.1. Cultures of Neurons from DRG Neurons, AHCs, and Schwann Cells

DRG neurons, AHCs, and Schwann cells were prepared as described previously [9]. Briefly, primary DRG neurons were resected and isolated from 4-week-old male Wister rats (Sankyo Labo Service Corporation Inc., Tokyo, Japan) using a previous method [42]. Under a microscope, 30 thoracic and lumbar DRGs per rat were dissected from the spinal column, and the spinal roots and peripheral nerve trunks were carefully removed. Dissected DRGs from 4-week-old male Wister rats were enzymatically disaggregated at 37 °C in Hank’s solution (5.4 mM KCl, 0.3 mM Na_2_HPO_4_·7H_2_O, 0.4 mM KH_2_PO_4_, 4.2 mM NaHCO_3_, 1.3 mM CaCl_2_, 0.5 mM MgCl_2_·6H_2_O, 0.6 mM MgSO_4_·7H_2_O, 137 mM NaCl, and 5.6 mM D-glucose) containing 0.05% collagenase (FUJIFILM Wako Pure Chemical Industry, Osaka, Japan) for 2 h and subsequently in Hank’s solution containing 0.05% trypsin and 0.02% ethylenediaminetetraacetic acid (EDTA) for 1 h. AHC neurons were resected from the spinal cord of 4-week-old male Wister rats, isolated enzymatically, and disaggregated at 37 °C in Hank’s solution containing 0.05% trypsin and 0.02% EDTA for 1 h. After washing, cells were plated onto 35 mm poly L-lysine-coated dishes (AGC Techno Glass, Shizuoka, Japan) at a density of 0.5~1 × 10^6^ cells/dish. Before use, DRG neurons and AHCs were identified by Western blot analysis for LDHB (ab75167; Abcam, Cambridge, UK) and Islet1/2 (bs-7337R; Bioss Inc., Woburn, MA, USA), respectively [9]. Rat Schwann cells were purchased from ScienCell Research Laboratories (Carlsbad, CA, USA).

### 4.2. Analysis of Apoptosis, Necrosis, and Necroptosis

To detect apoptosis, necrosis, and necroptosis caused by methylmercury (Sigma-Aldrich, Japan, Tokyo, Japan), DRG neurons were pretreated with or without necrostatin-1 (1 µM; Adipogen Life Sciences, San Diego, CA, USA) for 1 h in Dulbecco’s modified Eagle’s medium supplemented with 10% fetal bovine serum (Biosera, Kansas, MO, USA) and treated with methylmercury (0.5 µM) for 24 h in fresh medium. The cells were then suspended in a binding buffer containing annexin V-FITC (BioLegend, San Diego, CA, USA) and PI, followed by FACS analysis according to the manufacturer’s protocol. The FACS equipment and software used were a flow cytometer (FACSCalibur Flow Cytometer, Becton, Dickinson and Co., Franklin Lakes, NJ, USA) and FlowJo software v.10.7 (Becton, Dickinson and Co.), respectively. Separately, DRG neurons, AHCs, and Schwann cells were cultured at 37 °C until subconfluent and treated with methylmercury (0.5, 1, and 3 µM) for 24 h. After the treatment, the cells were stained with TUNEL (apoptotic cells) or 4′,6-diamidino-2-phenylindole (DAPI, nuclei) and observed under a Keyence BZ-9000 fluorescence microscope (Keyence, Osaka, Japan). Fluorescence intensity was calculated using ImageJ software v.1.53 (National Institutes of Health, Bethesda, MD, USA). To examine the involvement of TNF-α and its downstream signaling, DRG neurons were cultured in Dulbecco’s modified Eagle’s medium supplemented with 10% fetal bovine serum in 6-well culture plates and treated with methylmercury (0.5, 1, and 3 µM) for 24 h. After the treatment, secreted TNF-α was detected using a conditioned medium of DRG neurons treated with methylmercury. The cell layer was washed twice with calcium- and magnesium-free phosphate-buffered saline (Nissui Pharmaceutical, Tokyo, Japan) and lysed with sodium dodecyl sulfate (SDS) lysis buffer (50 mM Tris-HCl buffer [pH 6.8] containing 2% SDS and 10% glycerol). The cell lysate was incubated at 95 °C for 5 min. Protein concentration was determined using a bicinchoninic acid protein assay kit (Nacalai Tesque, Kyoto, Japan). The samples were incubated with 2-mercaptoethanol and bromophenol blue at 95 °C for 3 min. Protein samples were separated by SDS–polyacrylamide gel electrophoresis (PAGE) and transferred onto Immobilon-P transfer membranes (Merck Millipore, Billerica, MA, USA). The membrane was blocked with 5% skim milk in Tris-buffered saline containing Tween 20 (TBST; 20 mM Tris-HCl, 15 mM NaCl, and 0.1% Tween 20) or Blocking One solution (Nacalai Tesque) at room temperature for 1 h. The membrane was then washed with TBST and incubated with primary antibodies (Table 1) in 1% BSA-TBST or blocking solution at 4 °C overnight. After washing with TBST, the membrane was incubated with a horseradish peroxidase-conjugated secondary antibody in TBST at room temperature for 1 h. Immunoreactive bands were visualized using Chemi-Lumi One L (Nacalai Tesque) and recorded using a LAS-3000 device (Fujifilm, Tokyo, Japan). Band intensity was quantified using ImageJ software v.1.53.

### 4.3. Expression of TNF-α in Macrophage-like RAW264.7 Cells

Macrophage-like RAW264.7 cells were cultured in high-glucose Dulbecco’s modified Eagle’s medium (Nissui Pharmaceutical, Tokyo, Japan) supplemented with 10% fetal bovine serum in a humid atmosphere of 5% CO_2_. The cells were transferred to 6-well culture plates at 5.0 × 10^4^ cells/cm^2^, cultured for 12 h in Dulbecco’s modified Eagle’s medium supplemented with 10% fetal bovine serum, and incubated at 37 °C for 12 h in fresh high-glucose Dulbecco’s modified Eagle’s medium. The cells were then treated with methylmercury (1, 2, 3, 4, and 5 µM) for 12 h. The conditioned medium was harvested and used to determine TNF-α levels using TNF-α mouse ELISA kit quantikine (R&D systems, Minneapolis, MN, USA). The cell layer was washed twice with ice-cold CMF-PBS and scraped with the SDS lysis buffer. The cell lysate was used to determine DNA content using a fluorometric method [43]. The accumulation of TNF-α was expressed as pg/µg DNA. To determine TNF-α mRNA levels, the cells were treated with methylmercury (3 or 5 µM) for 4, 6, or 8 h in 6-well culture plates. The conditioned medium was discarded, and the cell layer was collected using QIAzol lysis reagent (Qiagen, Venlo, The Netherlands). The complementary DNA was synthesized using a high-capacity cDNA reverse transcription kit (Thermo Fisher Scientific, Waltham, MA, USA). Real-time polymerase chain reaction was performed using TaqMan Gene Expression Assay with 10 ng cDNA and 100 nM primers on a StepOnePlus RT-PCR system (Applied Biosystems, Waltham, MA, USA). The thermal cycling parameters were 95 °C for 10 min, followed by 40 cycles of 95 °C for 15 s and 60 °C for 1 min. The primer and probe sets for *GAPDH* (Mm99999915_g1) and *TNF-α* (Mm00443258_m1) were obtained from Thermo Fisher Scientific.

### 4.4. Expression of Phosphorylated IκBα and Nuclear Translocation of NF-κB

Macrophage-like RAW264.7 cells were treated with methylmercury (5 µM) for 3 or 6 h. To examine the nuclear translocation of NF-κB, cells were harvested in CMF-PBS and centrifuged at 4 °C for 5 min at 3000× *g*. The precipitates were separated into cytoplasm-rich and nucleus-rich fractions using NE-PER Nuclear and Cytoplasmic Extraction Reagent (Thermo Fisher Scientific), according to the manufacturer’s protocol. Briefly, after methylmercury treatment, the cells were suspended in ice-cold Cytoplasmic Extraction Reagent I, vortexed for 15 s, and incubated on ice for 10 min. Ice-cold Cytoplasmic Extraction Reagent II was then added, vortex for 5 s, and incubated on ice for 1 min. The solution was centrifuged at 15,000× *g* for 5 min at 4 °C to obtain the supernatant as the cytoplasm-rich fraction. The insoluble pellet was mixed with ice-cold Nuclear Extraction Reagent, followed by 4 repetitions of vortexing for 15 s and incubation on ice for 10 min. The solution was centrifuged at 15,000× *g* for 5 min at 4 °C to obtain the supernatant as the nucleus-rich fraction. Proteins in the cytoplasm-rich and nucleus-rich fractions were analyzed by SDS-PAGE and Western blotting as described above.

### 4.5. Hindlimb Extension Test

TNF-α KO mice (TNF-α^−/−^ mice) with a C57BL/6 × Sv129 background [44], 6–10-weeks old, were used. C57/6 mice (WT) were purchased from SLC (Hamamatsu, Shizuoka, Japan). All animals were housed in home cages under a 12 h/12 h light–dark cycle with ad libitum access to water and food. WT and TNF-α^−/−^ male mice were orally administered methylmercury (15 mg/kg/day) for 5 days, followed by no administration for 2 days. This cycle was repeated for 14 days. On day 9, after the first day of administration, the distances between the heels of the mice in the normal position were recorded.

### 4.6. Mercury Accumulation in Organs

WT and TNF-α^−/−^ male mice were orally administered methylmercury as above. The mice were anesthetized with carbon dioxide and each organ was surgically collected 9 days after the first day of administration. Tissue mercury (*m*/*z* = 202) was measured using ICP-MS (NexION 300S PerkinElmer, Waltham, MA, USA), as previously described [11].

### 4.7. Statistical Analysis

Statistical analyses were performed using Excel v.16.90 (Microsoft, Redmond, WA, USA) with the Statcel4 add-in (OMS, Tokyo, Japan). The data were expressed as the mean ± standard error. The statistical significance of the data was determined using one-way analysis of variance with the post-hoc Bonferroni/Dunn multiple test or Student’s *t*-test, as appropriate. Differences between groups were considered significant at *p* < 0.05.

## Figures and Tables

**Table 1:** Primary antibodies used in this study.

Antibody	Species	Clonality	Supplier	Code No
Caspase3	Rabbit	Polyclonal	Thermo Fisher Scientific	PA1-26426
Caspase8	Rabbit	Polyclonal	Novus Biologicals (Littleton, CO, USA)	NB-100-56116
TNF-α	Goat	Polyclonal	SantaCruz Biotechnology (Dallas, TX, USA)	sc-1351
TNFR1	Mouse	Monoclonal	SantaCruz Biotechnology	sc-8436
RIP3	Rabbit	Polyclonal	Novus Biologicals	NBP1-77299
Phospho-RIP3	Rabbit	Monoclonal	Abcam	ab195117
MLKL	Rat	Monoclonal	Merck	MABC604
Phospho-MLKL	Rabbit	Monoclonal	Abcam	ab196436
GAPDH	Mouse	Monoclonal	FUJIFILM Wako Pure Chemical Industry	#5A12
IκBα	Mouse	Monoclonal	Cell Signaling Technology (Danvers, MA, USA)	#4814
Phospho-IκBα	Mouse	Monoclonal	Cell Signaling Technology	#9246
NF-κB (p65)	Mouse	Monoclonal	Cell Signaling Technology	#6956
LaminA/C	Mouse	Monoclonal	SantaCruz Biotechnology	sc-7292

## Data Availability

The original contributions presented in the study are included in the article/Supplementary Materials; further inquiries can be directed to the corresponding authors.

## Supplementary Materials

The following supporting information can be downloaded from: https://www.mdpi.com/article/10.3390/ijms252111672/s1.

## References

[1] Eto K. (1997). Pathology of Minamata disease. Toxicol. Pathol..

[2] Hirooka T., Yamamoto C., Yasutake A., Eto K., Kaji T. (2013). Expression of VEGF-related proteins in cultured human brain microvascular endothelial cells and pericytes after exposure to methylmercury. J. Toxicol. Sci..

[3] Hirooka T., Yoshida E., Eto K., Kaji T. (2017). Methylmercury induces hyaluronan synthesis in cultured human brain microvascular endothelial cells and pericytes via different mechanisms. J. Toxicol. Sci..

[4] Du K., Hirooka T., Sasaki Y., Yasutake A., Hara T., Yamamoto C., Fujiwara Y., Shinoda Y., Fujie T., Katsuda S. (2023). Pathogenesis of selective damage of granule cell layer in cerebellum of rats exposed to methylmercury. J. Toxicol. Sci..

[5] Eto K., Tokunaga H., Nagashima K., Takeuchi T. (2002). An autopsy case of minamata disease (methylmercury poisoning)–pathological viewpoints of peripheral nerves. Toxicol. Pathol..

[6] Jacobs J.M., Carmichael N., Cavanagh J.B. (1977). Ultrastructural changes in the nervous system of rabbits poisoned with methyl mercury. Toxicol. Appl. Pharmacol..

[7] Su M., Kakita A., Wakabayashi K., Yamada M., Takahashi H., Ikuta F. (1997). Degeneration of spinal dorsal root ganglia in adult rats treated with methylmercury: Chronological observations on the cell bodies, centrally directed axons and presynaptic terminals. Neuropathology.

[8] Cao B., Lv W., Jin S., Tang J., Wang S., Zhao H., Guo H., Su J., Cao X. (2013). Degeneration of peripheral nervous system in rats experimentally induced by methylmercury intoxication. Neurol. Sci..

[9] Yoshida E., Aoki K., Sasaki Y., Izuhara H., Takahashi T., Fujiwara Y., Fujie T., Du K., Eto K., Shinoda Y. (2024). Comparative study of susceptibility to methylmercury cytotoxicity in cell types composing rat peripheral nerves: A higher susceptibility of dorsal root ganglion neurons. J. Toxicol. Sci..

[10] Shinoda Y., Ehara S., Tatsumi S., Yoshida E., Takahashi T., Eto K., Kaji T., Fujiwara Y. (2019). Methylmercury-induced neural degeneration in rat dorsal root ganglion is associated with the accumulation of microglia/macrophages and the proliferation of Schwann cells. J. Toxicol. Sci..

[11] Shinoda Y., Tatsumi S., Yoshida E., Takahashi T., Eto K., Kaji T., Fujiwara Y. (2019). Gene expression profiles in the dorsal root ganglia of methylmercury-exposed rats. J. Toxicol. Sci..

[12] Kaczmarek A., Vandenabeele P., Krysko D.V. (2013). Necroptosis: The release of damage-associated molecular patterns and its physiological relevance. Immunity.

[13] Geppert T.D., Whitehurst C.E., Thompson P., Beutler B. (1994). Lipopolysaccharide signals activation of tumor necrosis factor biosynthesis through the ras/raf-1/MEK/MAPK pathway. Mol. Med..

[14] Yoshida E., Kurita M., Eto K., Kumagai Y., Kaji T. (2017). Methylmercury promotes prostacyclin release from cultured human brain microvascular endothelial cells via induction of cyclooxygenase-2 through activation of the EGFR-p38 MAPK pathway by inhibiting protein tyrosine phosphatase 1B activity. Toxicology.

[15] Sen C.K., Packer L. (1996). Antioxidant and redox regulation of gene transcription. FASEB J..

[16] Wang N., Liang H., Zen K. (2014). Molecular mechanisms that influence the macrophage m1-m2 polarization balance. Front. Immunol..

[17] Hayden M.S., Ghosh S. (2008). Shared principles in NF-kappaB signaling. Cell.

[18] Nomura R., Takasugi N., Hiraoka H., Iijima Y., Iwawaki T., Kumagai Y., Fujimura M., Uehara T. (2022). Alterations in UPR Signaling by Methylmercury Trigger Neuronal Cell Death in the Mouse Brain. Int. J. Mol. Sci..

[19] Fujimura M., Usuki F. (2020). Pregnant rats exposed to low-level methylmercury exhibit cerebellar synaptic and neuritic remodeling during the perinatal period. Arch. Toxicol..

[20] Antunes Dos Santos A., Appel Hort M., Culbreth M., López-Granero C., Farina M., Rocha J.B., Aschner M. (2016). Methylmercury and brain development: A review of recent literature. J. Trace Elem. Med. Biol..

[21] Costa L.G., Aschner M., Vitalone A., Syversen T., Soldin O.P. (2004). Developmental neuropathology of environmental agents. Annu. Rev. Pharmacol. Toxicol..

[22] Harada M. (1995). Minamata disease: Methylmercury poisoning in Japan caused by environmental pollution. Crit. Rev. Toxicol..

[23] Delio D.A., Reuhl K.R., Lowndes H.E. (1992). Ectopic impulse generation in dorsal root ganglion neurons during methylmercury intoxication: An electrophysiological and morphological study. Neurotoxicology.

[24] Sakamoto M., Wakabayashi K., Kakita A., Hitoshi T., Adachi T., Nakano A. (1998). Widespread neuronal degeneration in rats following oral administration of methylmercury during the postnatal developing phase: A model of fetal-type minamata disease. Brain Res..

[25] Castoldi A.F., Barni S., Turin I., Gandini C., Manzo L. (2000). Early acute necrosis, delayed apoptosis and cytoskeletal breakdown in cultured cerebellar granule neurons exposed to methylmercury. J. Neurosci. Res..

[26] Ni L., Wei Y., Pan J., Li X., Xu B., Deng Y., Yang T., Liu W. (2022). Shedding new light on methylmercury-induced neurotoxicity through the crosstalk between autophagy and apoptosis. Toxicol. Lett..

[27] Wilke R.A., Kolbert C.P., Rahimi R.A., Windebank A.J. (2003). Methylmercury induces apoptosis in cultured rat dorsal root ganglion neurons. Neurotoxicology.

[28] Kuo T.C., Lin-Shiau S.Y. (2004). Early acute necrosis and delayed apoptosis induced by methyl mercury in murine peritoneal neutrophils. Basic. Clin. Pharmacol. Toxicol..

[29] Reyes-Becerril M., Angulo C., Sanchez V., Cuesta A., Cruz A. (2019). Methylmercury, cadmium and arsenic(III)-induced toxicity, oxidative stress and apoptosis in Pacific red snapper leukocytes. Aquat. Toxicol..

[30] Yang C.Y., Liu S.H., Su C.C., Fang K.M., Yang T.Y., Liu J.M., Chen Y.W., Chang K.C., Chuang H.L., Wu C.T. (2022). Methylmercury induces mitochondria- and endoplasmic reticulum stress-dependent pancreatic β-cell apoptosis via an oxidative stress-mediated JNK signaling pathway. Int. J. Mol. Sci..

[31] Usuki F., Fujita E., Sasagawa N. (2008). Methylmercury activates ASK1/JNK signaling pathways, leading to apoptosis due to both mitochondria- and endoplasmic reticulum (ER)-generated processes in myogenic cell lines. Neurotoxicology.

[32] Ceccatelli S., Daré E., Moors M. (2010). Methylmercury-induced neurotoxicity and apoptosis. Chem. Biol. Interact..

[33] Watanabe J., Nakamachi T., Ohtaki H., Naganuma A., Shioda S., Nakajo S. (2013). Low dose of methylmercury (MeHg) exposure induces caspase mediated-apoptosis in cultured neural progenitor cells. J. Toxicol. Sci..

[34] Chang S.H., Lee H.J., Kang B., Yu K.N., Minai-Tehrani A., Lee S., Kim S.U., Cho M.H. (2013). Methylmercury induces caspase-dependent apoptosis and autophagy in human neural stem cells. J. Toxicol. Sci..

[35] Sokolowski K., Falluel-Morel A., Zhou X., DiCicco-Bloom E. (2011). Methylmercury (MeHg) elicits mitochondrial-dependent apoptosis in developing hippocampus and acts at low exposures. Neurotoxicology.

[36] Cuello S., Goya L., Madrid Y., Campuzano S., Pedrero M., Bravo L., Cámara C., Ramos S. (2010). Molecular mechanisms of methylmercury-induced cell death in human HepG2 cells. Food Chem. Toxicol..

[37] Chen J., Kos R., Garssen J., Redegeld F. (2019). Molecular insights into the mechanism of necroptosis: The necrosome as a potential therapeutic target. Cells.

[38] Iwai-Shimada M., Takahashi T., Kim M.S., Fujimura M., Ito H., Toyama T., Naganuma A., Hwang G.W. (2016). Methylmercury induces the expression of TNF-α selectively in the brain of mice. Sci. Rep..

[39] Toyama T., Hoshi T., Noguchi T., Saito Y., Matsuzawa A., Naganuma A., Hwang G.W. (2021). Methylmercury induces neuronal cell death by inducing TNF-α expression through the ASK1/p38 signaling pathway in microglia. Sci. Rep..

[40] Liu H., Sidiropoulos P., Song G., Pagliari L.J., Birrer M.J., Stein B., Anrather J., Pope R.M. (2000). TNF-alpha gene expression in macrophages: Regulation by NF-kappa B is independent of c-Jun or C/EBP beta. J. Immunol..

[41] Schütze S., Wiegmann K., Machleidt T., Krönke M. (1995). TNF-induced activation of NF-kappa B. Immunobiology.

[42] Haratake M., Koga K., Inoue M., Fuchigami T., Nakayama M. (2011). Absorption and retention characteristics of selenium in dorsal root ganglion neurons. Metallomics.

[43] Kissane J.M., Robins E. (1958). The fluorometric measurement of deoxyribonucleic acid in animal tissues with special reference to the central nervous system. J. Biol. Chem..

[44] Taniguchi T., Takata M., Ikeda A., Momotani E., Sekikawa K. (1997). Failure of germinal center formation and impairment of response to endotoxin in tumor necrosis factor alpha-deficient mice. Lab. Investig..

