# Supplementary material for: Mechanisms Underlying Sensory Nerve-Predominant Damage by Methylmercury in the Peripheral Nervous System
Source: Int J Mol Sci. 2024 Oct 30;25(21):11672. doi: 10.3390/ijms252111672 (PMC11545846; doi:10.3390/ijms252111672)
Supplement: Supplementary file 1 [file ijms-25-11672-s001.zip › ijms-3237932-supplementary.pdf]

Supplemental material and method

siRNA transfection

Double-strand control small interfering RNA (siRNA) and Mouse p65 siRNA (Sence: UCUAUAGGAACGUGAAAGGGGdTdT, anti-sence: CCUUUCACGUUCCUAUAGAGGdTdT) were purchased from Greiner Bio-One GmbH (Kremsmünster, Austria). RAW264.7 cells were transfected with siRNA using Lipofectamine RNAiMAX transfection reagent (Thermo Fisher Scientific) in accordance with the manufacturer’s protocol.

Figure S1

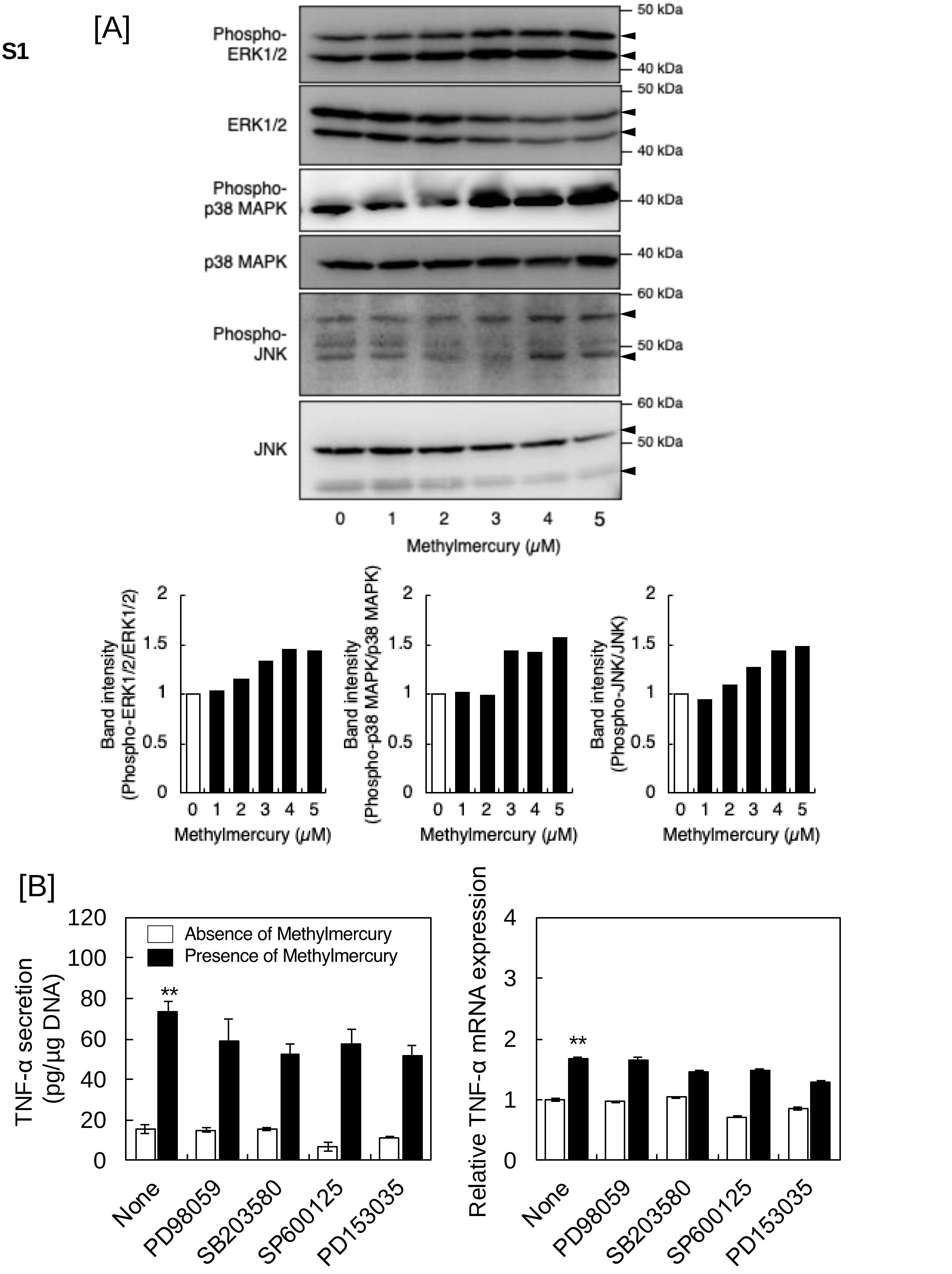

Figure S1. Possible involvement of phosphorylation of MAPKs in the expression of TNF-α in macrophage-like RAW264.7 cells after exposure to methylmercury. [A] Western blot images of phosphorylation of ERK1/2, p38 MAPK, and JNK in RAW264.7 cells treated with methylmercury. Arrows indicate the position of phospho-ERK1/2, ERK1/2, phospho-JNK, or JNK. [B] TNF-α secretion and mRNA levels in the cells pretreated with an ERK pathway inhibitor PD98059, p38 MAPK inhibitor SB203580, JNK inhibitor SP600125, or EGFR inhibitor PD153035 and then treated with methylmercury. Values are means ± S.E. of three technical replicates. **Significantly different from the corresponding control, $p < 0.01$.

Figure S2

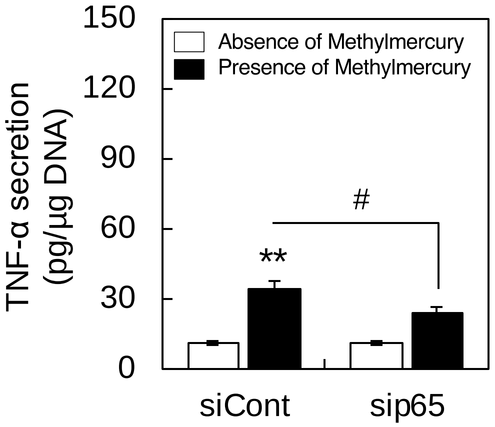

Figure S2. Possible involvement of p65, an NF-κB component, in the secretion of TNF-α from macrophage-like RAW264.7 cells after exposure to methylmercury. Secretion of TNF-α in conditioned medium of the cells transfected with control siRNA (siCont) or p65 siRNA (sip65) and then treated with methylmercury. Mouse p65 siRNA (Sence: UCUAUAGGAACGUGAAAGGGGdTdT, anti-sence: CCUUUCACGUUCCUAUAGAGGdTdT) . **Significantly different from the corresponding "absence of methylmercury", $p < 0.01$; #Significantly different from the corresponding "presence of methylmercury,"$p < 0.05$.
